# Comparison of Access Site-Related Complications and Quality of Life in Patients after Invasive Cardiology Procedures According to the Use of Radial, Femoral, or Brachial Approach

**DOI:** 10.3390/ijerph18116151

**Published:** 2021-06-07

**Authors:** Jan Roczniak, Wojciech Koziołek, Marcin Piechocki, Tomasz Tokarek, Andrzej Surdacki, Stanisław Bartuś, Michał Chyrchel

**Affiliations:** 1 Students’ Scientific Group at the Second Department of Cardiology, Jagiellonian University Medical College, 30-688 Cracow, Poland; roczniakjan@gmail.com (J.R.); wk26@interia.pl (W.K.); mpiech98@gmail.com (M.P.); 2 Department of Cardiology and Cardiovascular Interventions, University Hospital, 2 Jakubowskiego Street, 30-688 Cracow, Poland; tomek.tokarek@gmail.com (T.T.); surdacki.andreas@gmx.net (A.S.); stanislaw.bartus@uj.edu.pl (S.B.); 3 Center for Intensive Care and Perioperative Medicine, Faculty of Medicine, Jagiellonian University Medical College, 30-901 Cracow, Poland; 4 Second Department of Cardiology, Institute of Cardiology, Faculty of Medicine, Jagiellonian University Medical College, 30-688 Cracow, Poland

**Keywords:** periprocedural complications, quality of life, cardiovascular interventions

## Abstract

The radial approach (RA) is the most common in invasive cardiology, but depending on the clinical situation, the femoral approach (FA) and brachial approach (BA) are also used. The BA is associated with the highest odds of complications so it is used mainly if a first-choice approach fails. The aim of the study was to assess clinical outcomes after invasive cardiology procedures stratified by the use of the RA, FA, and BA, with a focus on access site-related complications, quality of life (QoL), and patients’ perspective. A total of 250 procedures (RA: 98; FA: 99; BA: 53) performed between 2013 and 2020 were retrospectively analyzed. Puncture site-related complications, vascular events, patient preferences, and QoL were assessed by the analysis of medical records and telephone follow-up using a proprietary questionnaire and the modified EQ-5D-3L questionnaire. Patients from the RA group received the smallest volume of contrast during a percutaneous coronary interventions (PCI) procedure (RA vs. FA vs. BA: 180 (150–240) mL vs. 200 (180–270) mL vs. 190 (100–200) mL, *p* = 0.045). The access site was changed most frequently in the procedures initiated from the RA (*p* < 0.04). Overall puncture site-related complications, especially local hematomas, occurred most commonly in the BA group (7.1, 14.1, and 24.5% for RA, FA, and BA, respectively, *p* = 0.01). During the index procedure, the access site was changed most frequently in procedures initiated from the RA (19.7, 8.5 and 0%, *p* = 0.04). The RA was indicated as an approach preferred by the patient for a hypothetical next procedure (87.9, 55.4, and 70.0% for subjects preferring the same approach out of patients who underwent a procedure by the RA, FA, and BA, respectively, *p* < 0.001). For the RA and FA, the prevalence of moderate or extreme access site-related problems in self-care decreased significantly (RA: *p* < 0.01, FA: *p* < 0.05) within 1 month after the index procedure (RA: 18.1, 4.2, and 1.4%; FA: 20.7, 11.1, and 9.6% periprocedurally, after 1 and 6 months, respectively). In contrast, for the BA these percentages were higher and a significant improvement (*p* < 0.05) was delayed until 6 months (54.6, 36.4, and 18.2% periprocedurally, after 1 and 6 months, respectively). In conclusion, compared to the BA and FA, the RA appears to be not only the safest, mainly due to the lowest risk of puncture site-related complications after coronary procedures but also represents a preferable approach from the patient’s perspective. Although overall post-procedural QoL outcomes did not differ significantly according to the access site, nevertheless, the BA was associated with more frequent self-care problems whose improvement was delayed until more than one month after the index procedure.

## 1. Introduction

The radial approach (RA) is nowadays the most widely used access site and is considered the safest one [1,2,3]. The RA offers a decrease in access site complications, such as local bleedings, and shortens hospitalization time, which implies reduced hospitalization costs [1,2,3,4]. 

However, depending on the clinical situation, the femoral approach (FA) and brachial approach (BA) are also used. The FA is still the most common mode of vascular access for coronarography in many countries, though the RA is on the rise [5]. The femoral artery is a large-caliber artery (allowing the use of larger-sized catheters), thereby remaining the preferable access site for many procedures such as transcatheter aortic valve replacement (TAVI), cardiac arrest invasive procedures, high-risk percutaneous coronary interventions (PCI), or implantation of intra-aortic balloon pump [5]. The BA is associated with the highest odds of complications so it is used mainly if a first-choice approach (RA or FA) fails [6,7,8]. A change of approach may occur due to catheterization failure because of anatomical variations or complex coronary narrowings, as well as a need to use a specific technique of angioplasty. 

In addition, patient’s personal preferences regarding vascular access (e.g., due to their profession) or the patient’s request may influence the type of approach used. Quality of life (QoL) analysis provides the basis for comparing different treatment options [9,10] and determining predictors of health benefits [11]. Although the impact of the vascular access site on QoL was discussed in the literature, it was focused merely on the RA and FA [12,13,14,15,16]. Therefore, our aim was to compare clinical outcomes after invasive cardiology procedures stratified by the use of the RA, FA, and BA, with a focus on access site-related complications, quality of life (QoL), and patients’ perspective.

## 2. Materials and Methods

### 2.1. Patients

The study group consisted of 250 catheterization procedures, mostly PCI or coronarographies, performed between 2013 and 2020 in our center. The interventions were performed on 208 patients, 172 of whom underwent one procedure, and 36 had two to four procedures, during either a single or separate hospitalizations. The inclusion criterion was cardiac catheterization via the RA, FA, or BA. Manual compression was used as a method of artery closure at the end of the procedure in all of the analyzed cases. The exclusion criterion was a lack of complete data in available medical records. 

### 2.2. Data Analysis

Patients were divided into three groups according to the approach that was used to carry out the procedure. To assess complications, cases were analyzed “as treated”, according to the final approach, whereas the necessity to change the initial puncture site was analyzed “as intended”, according to the approach via which the procedure was initiated. In order to increase the number of subjects in the BA group, peripheral transluminal angioplasty (PTA), arteriography, and valvuloplasty procedures were also included in the BA group to compare the puncture-site complications. As to vascular complications, only PCI procedures were taken into account because coronarography without PCI was not associated with any vascular complications. 

**Figure 1:**
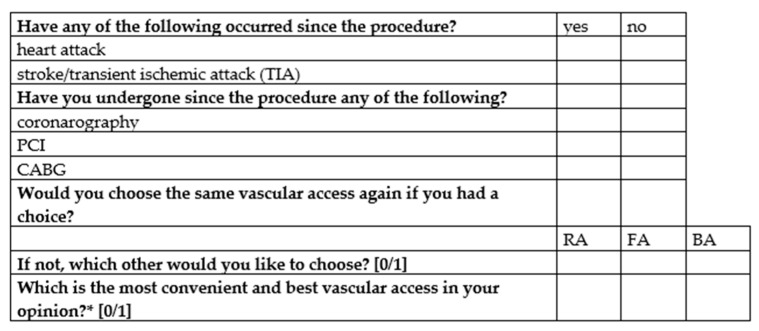
A proprietary telephone questionnaire assessing long-term complications of the procedure and the patient’s personal preferences regarding vascular access. * a question for patients who underwent procedures with the use of more than one access, CABG—coronary artery bypass grafting, PCI—percutaneous coronary intervention.

Data were collected retrospectively, for the RA and FA, approximately 100 of the most recent consecutive cases each, whereas for the BA, we collected all the cases from the unit’s archive. Among the 100 most recent FA procedures, there were procedures other than coronarography or PCI. They were included in the study to increase the similarity to the BA group. The 100 most recent cases with the use of RA did not include other procedures. The next stage of the study was the telephone clinical follow-up to estimate the QoL outcomes. For this purpose, two questionnaires were used. The first, proprietary one (Figure 1), estimated long-term complications of the procedure and the patient’s personal preferences regarding vascular access, while the second one was a Polish version of a script for telephone administration, the EQ-5D-3L (Poland (Polish) 2005 EuroQol Group EQ-5D™, The EuroQol Research Foundation, Rotterdam, The Netherlands). The EQ-5D-3L questionnaire was used with the permission of the EuroQol Research Foundation. The EQ-5D-3L questionnaire included questions asked retrospectively with respect to their perioperative state, one month after and six months after the index procedure. Additionally, the visual analog scale (VAS) (EQ-VAS) was filled by the researchers after having asked patients about their own subjective assessment of their global health status on a scale from 0 to 10.

The exclusion criterion from the telephone clinical follow-up was the date of the procedure before 2017 so that 32 out of 250 cases were excluded. All of them belonged to the BA group, therefore the number of BA procedures included in the follow-up decreased to 21. For the remaining 218, a maximum of three contact attempts was made, which was successful in 166 cases. Fifteen interviews were excluded due to patient death (4 from the RA group, 8 from the FA group, and 3 from the BA group), while consent to the interview was obtained in 146 out of 151 remaining cases. The final analysis encompassed 72, 63, and 11 interviews related to interventions performed via the RA, FA, and BA, respectively. 

The study was carried out in agreement with ethical principles for clinical research based on the Declaration of Helsinki. The study protocol was approved by the Jagiellonian University Bioethical Committee (approval No.: 1072.6120.332.2020 issued on 16 December 2020). 

### 2.3. Statistical Analysis

Standard descriptive statistics were used to describe the data. Categorical variables are presented as percentages, with continuous variables as mean values and standard deviations (SD) for normally distributed data or medians with interquartile range (Q1–Q3) for non-normally distributed data. Data normality was assessed using the Shapiro-Wilk test for samples smaller than 50 or Kolmogorov-Smirnov test for samples greater than 50. 

Quantitative variables with normal distribution were compared using a one-way ANOVA test with posthoc Tukey’s test for 3 groups or t-Student test for 2 groups. Non-normally distributed quantitative variables were compared using Kruskal-Wallis ANOVA with posthoc Dunn’s test for 3 groups or Mann-Whitney-Wilcoxon U test for 2 groups. Repeated measurements with a non-normal distribution were estimated by Friedman’s ANOVA or Wilcoxon signed-rank test. Categorical variables were compared using Pearson’s chi-square test. 

The level of statistical significance was assumed set at a *p*-value < 0.05. All analyses were carried out with the software TIBCO Statistica (version 13. Palo Alto, CA, USA).

## 3. Results

### 3.1. Patients’ Characteristics

The study population consisted of 208 patients who underwent a total of 250 procedures. One procedure was performed in 172 patients, while 36 subjects had at least two interventions. There were 98 interventions assigned to the RA group, 99 to the FA group, and 53 to the BA group. Characteristics of patients are presented in Table 1. In some cases, there were two causes of a procedure, especially with regard to chronic total occlusion (CTO) in patients with stable or unstable angina; therefore, total percentages may be greater than 100%. The index intervention concerned most commonly one vessel, mainly left anterior descending artery (47.7%), followed by right coronary artery (39.4%), left circumflex artery, left main coronary artery (26.2%), and other vessels. Again, respective cumulative percentages can exceed 100% because some procedures affected more than one vessel. 

Median radiation dose was significantly higher (*p* < 0.001) in PCI procedures than in coronarography (Table 2). No vascular complications occurred after coronarography, whereas 18.6% of PCI procedures resulted in vascular complications (*p* < 0.001). No significant differences were observed between coronarography and PCI in terms of puncture site-related complications (*p* = 0.15) (Table 2). 

The FA was the most common access chosen to treat CTO (RA: 34.3%; FA: 65.7%). However, the proportion of successful PCI in CTO was similar for the RA (50%) and FA (46.4%).

### 3.2. Complications

Overall, puncture site-related complications occurred most commonly in the procedures carried out via the BA (Table 3). Access site was changed most frequently in the procedures initiated from the RA (*p* < 0.001). In particular, local hematomas occurred most commonly in the BA group (*p* = 0.007) (Table 3). No significant differences were observed in terms of other complications: pseudoaneurysm, arteriovenous fistula, blood transfusion related to blood loss, or bleeding from the puncture site (Table 3). Retroperitoneal bleeding, a complication strictly limited to the FA, was observed in 2.0% of patients in the FA group. 

There were no differences in the occurrence of vascular complications in the PCI procedures, nor in any specific complication, such as coronary dissection, coronary perforation, no-reflow or slow-flow phenomenon, periprocedural myocardial infarction, or cardiac tamponade (Table 3). 

The access site was changed most frequently in the procedures initiated by the RA (RA: 23.6%; FA: 9.6%; BA: 0%, *p* < 0.001). However, this result can be biased due to the patients’ selection, since all the BA procedures present in the archive have been chosen. BA procedures often result from catheterization failure via the RA, so this choice could exaggerate the rate of necessity to change access site in procedures initiated from the RA. To minimize this hypothetical bias, another comparison was performed with the BA group restricted to the same treatment time span (2019–2020) as the RA and FA (RA: 98; FA: 99; BA: 13). This comparison has shown a similar relationship, with a slightly smaller rate of access change via the RA due to the elimination of some procedures converted to the BA after an unsuccessful attempt with the use of the RA (RA: 19.7%; FA: 8.5%; BA: 0%, *p* = 0.04) (Table 3). No differences were observed in the proportion of unsuccessful PCI (*p* = 0.4) (Table 3). 

Patients from the RA group received the smallest volume of contrast during a PCI procedure (*p* = 0.045) (Table 3). No differences were observed in the volume of contrast received during a coronarography, neither in the radiation dose received at a PCI nor in the radiation dose during a coronarography (Table 3). 

There were no periprocedural deaths or strokes in the present study and no patients were referred for emergency bypass surgery. There was a slightly longer hospitalization in the BA group (*p* = 0.06) (Table 3). 

Due to some doubts concerning procedures with approach conversion, further analysis with exclusion of these cases has been conducted (RA: 94; FA: 65; BA: 46). The analysis provided similar results – occurrence of hematomas and site complications in general was the most common in the BA group (any complication: 19.6%, 7.5%, 13.9%, *p* = 0.11; hematoma: 17.8%, 6.4%, 10.8%, *p* = 0.12; BA, RA, FA respectively). The *p*-value was not significant which can be explained by the decrease in the analyzed sample size.

Similarly, to avoid controversy with the inclusion of peripheral procedures, another analysis with exclusion of those cases was conducted (RA: 98; FA: 93; BA: 41). Again, the results were similar (any complication: 20%, 7%, 14%, *p* = 0.1; hematoma: 18%, 6%, 10%, *p* = 0.11; BA, RA, FA respectively). This exclusion greatly restricted the number of cases in the BA group, which can explain a non-significant *p*-value.

### 3.3. Follow-Up

The time of collecting data via the telephone questionnaire ranged from six months to three years after the index procedure was performed in 72, 63, and 11 patients treated via the RA, FA, and BA, respectively. According to Question 1 and Question 2 of the proprietary telephone questionnaire, there were no significant intergroup differences in the incidence of heart attack (*p* = 0.13), stroke or transient ischemic attack (*p* = 0.5), as well as in the proportion of subjects undergoing repeated coronarography (*p* = 0.7) or coronary revascularization (*p* = 0.4 for PCI and *p* = 0.8 for CABG). 

Question number 3 regarding the choice of the same vascular access for a hypothetical next procedure was answered in 124 cases (RA: 58; FA: 56; BA: 10), while in the remaining 22 cases, the patients answered that they would rely on the choice of a physician or expressed a neutral attitude to this issue. The desire to choose the same approach was expressed by 87.9% of the RA group (*n* = 51), 70.0% of the BA group (*n* = 7), and only 55.4% in the FA group (*n* = 31) (*p* < 0.001). Out of 35 negative answers to Question 3, another preferred access, as an answer to Question 4, was indicated 27 times. All the patients from the RA group (*n* = 5) and BA group (*n* = 3) would choose the FA for the next procedure, whereas the RA was the only choice in the FA group (*n* = 19) (*p* < 0.001). Patients who underwent procedures with the use of more than one access (*n* = 44) considered the RA (70.4%) as the most convenient approach, others indicated the FA (29.6%), and no one designated the BA. 

**Figure 2:**
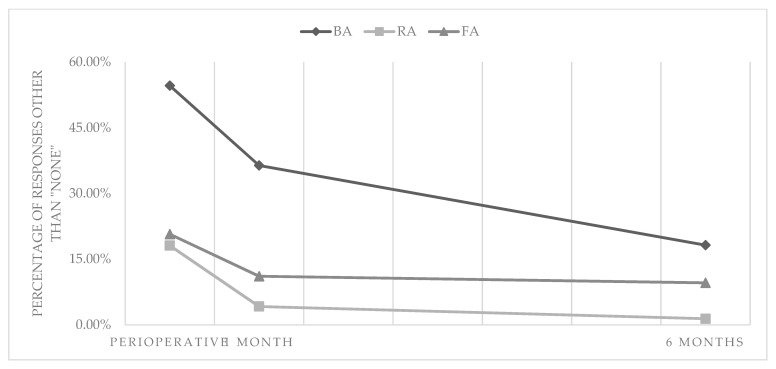
Percentages of responses other than "none" to the question from the EQ-5D-3L questionnaire regarding problems with self-care and their change over time.

Median values of the EQ-5D-3L questionnaire are shown in Table 4 and detailed answers in Table 5. Results of the EQ-VAS are presented in Table 6. The statistically significant intergroup differences were observed only for self-care problems, indicating the association of worse outcomes with the BA. For the RA and FA, the prevalence of moderate or extreme access site-related problems in self-care decreased significantly (RA: *p* < 0.01, FA: *p* < 0.05) within 1 month after the index procedure (RA: 18.1, 4.2, and 1.4%; FA: 20.7, 11.1, and 9.6% periprocedurally after 1 and 6 months, respectively). In contrast, for the BA, these percentages were higher and a significant improvement (*p* < 0.05) was delayed until 6 months (54.6, 36.4, and 18.2% periprocedurally after 1 and 6 months, respectively) (Figure 2).

## 4. Discussion

Our salient finding was the lowest incidence of puncture site-related complications, especially local hematomas, after coronary procedures performed via the RA, while the BA was associated with the highest respective risk and the FA with intermediate values. Additionally, the volume of contrast media during PCI was lowest for the RA. There were no significant intergroup differences in overall post-procedural QoL by the access site. However, compared to the RA and FA, the BA was associated with a markedly increased incidence of self-care problems and their improvement was delayed until several months after discharge. Finally, the RA was a preferable approach from the patients’ perspective with regard to a hypothetical next procedure. 

It is well-recognized that the BA is accompanied by the greatest risk of complications [6,7,8]. Additionally, in our research, the BA was associated with the highest risk of a puncture site hematoma, which occurred in 23% of patients. In comparison to other studies, hematomas also occurred most commonly in the BA group or were a frequent complication related to the BA, nevertheless, it was generally less common in previous reports compared to the present study, ranging from 1% to 14% [7,8,17,18]. According to Otsuka et al. [17], pseudoaneurysm was also most common in the BA group (1.3%) versus the RA or FA groups, which was also observed in our study (1.9%), although the respective difference did not reach statistical significance, presumably owing to the number of study subjects. Although often mentioned as a complication of BA interventions [7,8,19], brachial artery thrombosis did not occur in any of the cases in our study.

Baker and Baker [20] reported that the rate of bleeding complications was about 3–6% with the FA, with many patients developing retroperitoneal bleeding and up to 1% of those patients requiring blood transfusions [20]. Additionally, the rate of major bleeding assessed by Otsuka et al. was highest for the FA and, consequently, blood transfusion rates were highest in that group [17]. In our analysis, the FA was associated with only one significant bleeding and local hematoma occurred in 11% cases from the FA group, while blood transfusion was required most commonly in the FA group, albeit below the level of significance. As minor bleedings are often not recorded in the documentation, this methodological issue could contribute to some differences between the compared studies. With regard to retroperitoneal bleeding, a complication specific for the FA, its incidence (2%) was similar to that reported by Otsuka et al. [17]. 

In a systematic review, Mitchel et al. [21] found that the RA was almost 5-fold more likely to be associated with catheterization failure than the FA. In agreement with that observation, we have shown that the necessity to change the initial access site was highest for the RA. This is consistent with the notion that a non-RA access site, the FA and especially the BA, is chosen by the operator mainly as an alternative approach in case of problems related to the primary access site. 

Patients undergoing catheter procedures often prefer the RA [4,13,15,16,22], so it might appear to be associated with better QoL outcomes. However, the results of our study did not prove RA superiority over other access sites with respect to overall QoL. Nevertheless, the BA, compared to the RA and FA, seems to be associated with more problems with self-care that resolve more slowly than with the RA or FA. Similar observations have not yet been reported in the literature. On the other hand, in the BA group, self-care problems persisted for six months after the index procedure, which may indicate that the results could be affected by other factors than solely access site. 

There are discordant results with regard to the comparison of postoperative QoL between the RA and FA. In 200 subjects, Cooper et al. [4] compared QoL 1 day and 1 month after cardiac catheterization using the RA or FA and revealed better QoL for the RA. An identical conclusion was drawn from a more recent study which estimated QoL 1 day after PCI in about 1500 patients [12]. A significantly better QoL (assessed from 2 h to 4 days after PCI) with the RA was also reported in a study comprising about 100 patients with ST-segment elevation myocardial infarction [13]. Nevertheless, some other studies argue against an unequivocal difference in QoL in favor of the RA. Reddy et al. [14] found no statistical difference of QoL after the RA and FA in 75 patients followed for 1–7 days after diagnostic cardiac catheterization [15]. Likewise, Hess et al. [15], who assessed QoL at discharge and after 30 days post-cardiac catheterization among about 300 women, found no differences in post-procedural QoL according to the access site [15]. A similar finding was described in a study that estimated QoL 1–4 days after intervention in approximately 140 patients [16]. The results of these three reports [14,15,16] appear consistent with our results, i.e., the lack of significant differences in the overall postoperative QoL study between the RA and FA. 

## 5. Limitations

This study has certain limitations. Firstly, the procedures through the BA were performed in 2013–2020, while those assigned to the RA and FA are from 2019–2020. Secondly, a large number of procedures performed with the FA or BA accesses constitute conversion from another access, most often RA. The QoL outcomes are based on a follow-up questionnaire survey, which can be subjective and biased, as impairments were assessed retrospectively by the patients. Moreover, records of follow-up body examinations or laboratory analyses are not available. Finally, the number of the BA group in QoL follow-up is quite small. 

## 6. Conclusions

In conclusion, compared to the BA and FA, the RA appears to be not only the safest, mainly due to the lowest risk of puncture site-related complications after coronary procedures but it also represents the preferred approach from the patient’s perspective. Although overall post-procedural QoL outcomes did not differ significantly according to the access site, nevertheless, the BA was associated with more frequent self-care problems with a delayed improvement until more than one month after the index procedure. 

## Figures and Tables

**Table 1:** Characteristics of patients and procedures.

Category	Subcategory	BA *n* = 53	RA *n* = 98	FA *n* = 99	*p* Value	All Patients
Medical history	Hypertension	88.7%	86.3%	95.2%	0.062	90.2%
	Hypercholesterolemia	78.9%	81.1%	80.2%	0.950	80.2%
	Previous PCI	44.2%	46.3%	57.5%	0.193	50.2%
	Previous MI	53.9%	43.2%	46.8%	0.463	46.9%
	Nicotinism	55.8%	41.1%	42.6%	0.196	44.8%
	Diabetes	32.1%	39.0%	36.8%	0.706	36.6%
Reasonsfor the procedure	Stable angina	41.5%	56.7%	48.5%	0.187	50.2%
	NSTEMI	28.3%	17.5%	21.2%	0.305	21.3%
	Unstable angina	11.3%	13.4%	11.1%	0.871	12.0%
	STEMI	7.55%	8.25%	7.07%	0.953	7.6%
Type of procedure	Coronarography only	22.6%	51.0%	25.3%		34.8%
	PCI:	54.7%	49.0%	68.7%		58.0%
	1 vessel	35.9%	34.7%	51.5%		41.6%
	2 vessels	5.7%	9.2%	9.1%		8.4%
	3 vessels	7.6%	1.0%	4.0%		3.6%
	4 vessels	0%	1.0%	0%		0.4%
	Coronary artery bypass	3.7%	1.0%	2.0%		2.0%
	undefined	1.9%	2.1%	2.1%		2.0%
	Others:	22.6%	0%	6.1%		7.2%
	PTA	20.7%		0%		4.4%
	valvuloplasty	1.9%		5.1%		2.0%
	angiography	0%		1.0%		0.4%

CTO—chronic total occlusion, MI—myocardial infarction, NSTEMI—non-ST-elevation myocardial infarction, PCI—percutaneous coronary intervention, PTA—percutaneous transluminal angioplasty, STEMI—ST-elevation myocardial infarction.

**Table 2:** Comparisons between PCI and coronarography.

Category	PCI *n* = 145	Coronarography *n* = 87	*p* Value
**Any vascular complication**	**18.6%**	**0%**	**<0.001**
Any puncture site complication	14.5%	8.1%	0.15
**Radiation dose [Gy]**	**0.603 (0.363–1.076)**	**0.180 (0.121–0.306)**	**<0.001**
**Contrast volume [mL]**	**200 (150–250)**	**100 (60–120)**	**<0.001**

Statistical significance (*p* < 0.05) is marked in bold. PCI—percutaneous coronarography intervention.

**Table 3:** Comparison of the frequency of complications according to vascular access site.

Category	Complication or Characteristic	BA	RA	FA	*p*-Value	All Patients
Puncture site-related (all procedures)	number	53	98	99		250
	**Access change**	**0.0%**	**19.7%**	**8.5%**	**0.04**	**14.5%**
	**Any puncture site-related** **complication**	**24.5%**	**7.1%**	**14.1%**	**0.01**	**13.6%**
	**Local hematoma**	**23.1%**	**6.1%**	**10.1%**	**0.007**	**11.2%**
	Pseudoaneurysm	1.9%	0.0%	1.0%	0.4	0.8%
	Arteriovenous fistula	0.0%	0.0%	0.0%	>0.9	0.0%
	Blood transfusion	3.9%	1.0%	5.1%	0.8	2.8%
	Bleeding	3.8%	0.0%	1.0%	0.12	1.2%
	Retroperitoneal hemorrhage	-	-	2.0%	-	-
Vascular (PCI only)	number	29	48	68		145
	Any vascular complication	10.3%	14.6%	19.1%	0.5	15.9%
	Coronary dissection	3.6%	12.5%	7.4%	0.3	8.3%
	Coronary perforation	0.0%	0.0%	5.9%	0.10	2.8%
	No-reflow or slow-flow	3.5%	0.0%	2.9%	0.5	2.1%
	Periprocedural MI	0.0%	2.1%	4.4%	0.5	2.8%
	Periprocedural stroke	0.0%	0.0%	0.0%	>0.9	0.0%
	Cardiac tamponade	0.0%	0.0%	1.5%	0.6	0.7%
	Unsuccessful PCI	16.0%	9.5%	24.2%	0.4	17.8%
Other	Hospitalization length [days]	6 (3–10)	5 (5–7)	5 (3–9)	0.06	5 (3–8)
	**Contrast volume during PCI [ml]**	**190** **(100–200)**	**180** **(150–240)**	**200** **(180–270)**	**0.045**	**200** **(150–250)**
	Contrast volume at coronarography [mL]	150 (80–150)	90 (60–100)	100 (75–150)	0.10	100 (60–120)
	Radiation dose at a PCI [Gy]	0.381 (0.227–1.100)	0.488 (0.340–1.020)	0.719 (0.457–1.160)	0.09	0.603 (0.363–1.076)
	Radiation dose at a coronarography [Gy]	0.360 (0.120–0.381)	0.180 (0.126–0.247)	0.202 (0.110–0.314)	0.7	0.180 (0.121–0.306)

Statistical significance (*p* < 0.05) is marked in bold. The data in the first row come from the BA time span restricted to the same span as the RA and FA, whereas the other rows come from the whole period. CTO—chronic total occlusion, MI—myocardial infarction, NSTEMI—non-ST-elevation myocardial infarction, PCI—percutaneous coronary intervention, PTA—percutaneous transluminal angioplasty, STEMI—ST-elevation myocardial infarction.

**Table 4:** EQ-5D-3L Questionnaire overall results.

Time Point	BA (*n* = 11)	RA (*n* = 72)	FA (*n* = 63)	*p* Value
Perioperative	2 (1–3)	2 (1–3)	1 (1–3)	0.7
1 month	1 (0–3)	0 (0–2)	0 (0–2)	0.3
6 months	0 (0–2)	0 (0–1)	1 (0–2)	0.13

Data are shown as medians (interquartile range). Maximal total score = 10 (5 questions; 0–2 points each); a lower score corresponds to better QoL outcomes.

**Table 5:** Specific answers from the EQ-5D-3L Questionnaire.

Time Point	Type of Problems	BA (*n* = 11)	RA (*n* = 72)	FA (*n* = 63)	*p* Value
Periprocedural	Mobility				
	none	72.7%	79.2%	71.4%	
	moderate	9.1%	9.7%	17.5%	0.6
	extreme	18.2%	11.1%	11.1%	
	**Self-care**				
	**none**	**45.5%**	**81.9%**	**79.4%**	
	**moderate**	**45.5%**	**12.5%**	**14.3%**	**0.03**
	**extreme**	**9.1%**	**5.6%**	**6.4%**	
	Usual activities				
	none	27.3%	62.5%	49.2%	
	moderate	45.5%	26.4%	38.1%	0.05
	extreme	27.3%	11.1%	12.7%	
	Pain or discomfort				
	none	81.8%	50.0%	50.8%	
	moderate	9.1%	43.1%	42.9%	0.2
	extreme	9.1%	6.9%	6.4%	
	Anxiety or depression				
	none	81.8%	61.1%	66.7%	
	moderate	9.1%	38.9%	25.4%	0.6
	extreme	9.1%	0.0%	7.9%	
1 month	Mobility				
	none	81.8%	87.5%	85.7%	
	moderate	9.1%	12.5%	14.3%	0.8
	extreme	9.1%	0.0%	0.0%	
	**Self-care**				
	**none**	**63.6%**	**95.8%**	**88.9%**	
	**moderate**	**27.3%**	**4.2%**	**7.9%**	**0.003**
	**extreme**	**9.1%**	**0.0%**	**3.2%**	
	Usual activities				
	none	45.5%	73.6%	68.3%	
	moderate	36.4%	16.7%	23.8%	0.2
	extreme	18.2%	9.7%	7.9%	
	Pain or discomfort				
	none	90.9%	86.1%	71.4%	
	moderate	0.0%	13.9%	27.0%	0.07
	extreme	9.1%	0.0%	1.6%	
	Anxiety or depression				
	none	81.8%	76.4%	77.8%	
	moderate	9.1%	23.6%	17.5%	>0.9
	extreme	9.1%	0.0%	4.8%	
6 months	Mobility				
	None	100.0%	90.3%	79.4%	
	Moderate	0.0%	8.3%	19.1%	0.08
	extreme	0.0%	1.4%	1.6%	
	**Self-care**				
	**none**	**81.8%**	**98.6%**	**90.5%**	
	**moderate**	**18.2%**	**1.4%**	**6.4%**	**0.03**
	**extreme**	**0.0%**	**0.0%**	**3.2%**	
	Usual activities				
	none	63.6%	80.6%	69.8%	
	moderate	27.3%	15.3%	23.8%	0.25
	extreme	9.1%	4.2%	6.4%	
	Pain or discomfort				
	none	81.8%	84.7%	76.2%	
	moderate	18.2%	15.3%	23.8%	0.45
	extreme	0.0%	0.0%	0.0%	
	Anxiety or depression				
	none	90.9%	84.7%	79.4%	
	moderate	0.0%	15.3%	14.3%	0.5
	extreme	9.1%	0.0%	6.4%	

Statistical significance (*p* < 0.05) is marked in bold.

**Table 6:** EQ-VAS (Visual Analogue Scale) results.

Time Point	BA (*n* = 11)	RA (*n* = 72)	FA (*n* = 63)	*p* Value
Periprocedural	7 (3–8)	6 (5–8)	6 (5–8)	0.9
1 month	8 (6–8)	7 (6–8)	7 (6–8)	0.9
6 months	8 (7–8)	8 (6–9)	8 (5–9)	0.9

Data are shown as median (interquartile interval). Maximal score = 10; a higher score corresponds to a better subjective health assessment.

## Data Availability

The data presented in this study are available on a reasonable request from the corresponding author.
